# Possible Susceptibility Genes for Intervention against Chemotherapy-Induced Cardiotoxicity

**DOI:** 10.1155/2020/4894625

**Published:** 2020-10-13

**Authors:** Xinyu Yang, Guoping Li, Tao Yang, Manke Guan, Na An, Fan Yang, Qianqian Dai, Changming Zhong, Changyong Luo, Yonghong Gao, Saumya Das, Yanwei Xing, Hongcai Shang

**Affiliations:** ^1^Guang'anmen Hospital, China Academy of Chinese Medical Sciences, Beijing 100053, China; ^2^Key Laboratory of Chinese Internal Medicine of the Ministry of Education, Dongzhimen Hospital Affiliated to Beijing University of Chinese Medicine, Beijing 100700, China; ^3^Cardiovascular Research Center, Massachusetts General Hospital, Harvard Medical School, Boston, MA 02114, USA; ^4^Beijing University of Chinese Medicine, Beijing 100029, China

## Abstract

Recent therapeutic advances have significantly improved the short- and long-term survival rates in patients with heart disease and cancer. Survival in cancer patients may, however, be accompanied by disadvantages, namely, increased rates of cardiovascular events. Chemotherapy-related cardiac dysfunction is an important side effect of anticancer therapy. While advances in cancer treatment have increased patient survival, treatments are associated with cardiovascular complications, including heart failure (HF), arrhythmias, cardiac ischemia, valve disease, pericarditis, and fibrosis of the pericardium and myocardium. The molecular mechanisms of cardiotoxicity caused by cancer treatment have not yet been elucidated, and they may be both varied and complex. By identifying the functional genetic variations responsible for this toxicity, we may be able to improve our understanding of the potential mechanisms and pathways of treatment, paving the way for the development of new therapies to target these toxicities. Data from studies on genetic defects and pharmacological interventions have suggested that many molecules, primarily those regulating oxidative stress, inflammation, autophagy, apoptosis, and metabolism, contribute to the pathogenesis of cardiotoxicity induced by cancer treatment. Here, we review the progress of genetic research in illuminating the molecular mechanisms of cancer treatment-mediated cardiotoxicity and provide insights for the research and development of new therapies to treat or even prevent cardiotoxicity in patients undergoing cancer treatment. The current evidence is not clear about the role of pharmacogenomic screening of susceptible genes. Further studies need to done in chemotherapy-induced cardiotoxicity.

## 1. Introduction

Cancer therapeutics have seen tremendous progress in recent years [1, 2] and have revolutionized the treatment strategies and outcomes of some types of cancer [3]. These novel therapeutic strategies target specific molecular entities implicated in disease pathogenesis. Advances in cancer treatment have improved the survival rates of cancer patients, but they have also increased morbidity and mortality due to side effects [4, 5], in particular, cardiovascular complications, including hypertension, arrhythmias, left ventricular (LV) dysfunction, and HF, which can manifest many years after the completion of chemotherapy [6]. For example, regardless of the infusion rate [7], maximum cumulative doses [8], and alternative drugs [9] to reduce heart injury, the incidence of cardiotoxicity caused by anthracyclines is 9% to 18% [10, 11]. Within 2 years of HF, patients have a mortality rate of 60%, an extremely poor prognosis [12]. Further, the incidence of myocarditis with checkpoint inhibitors can be as high as 13.9% [13]. As a result, cancer patients often suffer from a variety of cardiotoxicities induced by treatment, which can result in substantial adverse impact on their emotional, economic, and social well-being [14, 15]. Unfortunately, the mechanisms underlying chemotherapy-induced cardiotoxicity remain poorly understood.

**Figure 1:**
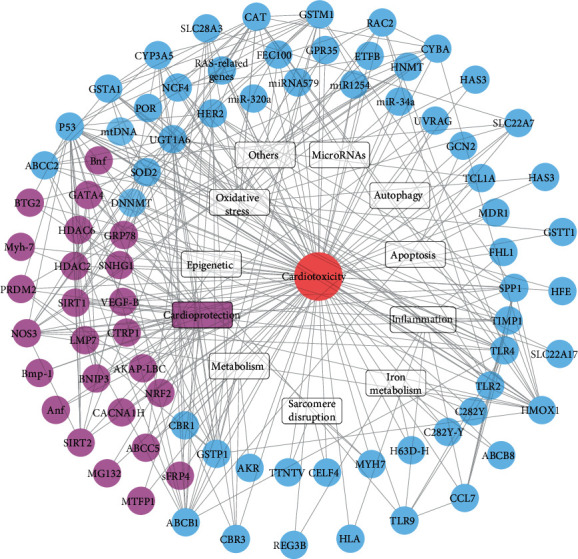
Graph of the protein network comprising combinations based on genetic studies that indicates the protective targets in the chemotherapy-induced cardiotoxicity.

Although clinical and demographic factors may increase the susceptibility of some individuals to the risk and severity of toxicity, individual differences in toxicity manifestations are considerable, exacerbating these toxicities. Genetics, therefore, could provide insights into the mechanism for toxicity induced by chemotherapy. The identification of genetic biomarkers able to predict whether a patient is at risk of developing cardiac dysfunction induced by chemotherapy will minimize cardiotoxicities during cancer treatment, through the administration of cardioprotective drugs or the use of optimized cancer therapies. Data from studies on genetic defects and pharmacological interventions have suggested that many molecules, primarily those regulating oxidative stress, inflammation, autophagy, apoptosis, and metabolism, contribute to the pathogenesis of cardiotoxicity induced by chemotherapy. In this article, we review the progress made in genetic research to elucidate the molecular mechanisms of chemotherapy-induced cardiotoxicity. Furthermore, a network of functionally related proteins from a STRING database [16] (Figure 1) was established to determine whether these targets play a role in the prediction of or protection against chemotherapy-induced cardiotoxicity. We propose a variety of cardioprotective mechanisms and provide insights for the development of therapies to reduce, or even cure, the cardiotoxicity induced by chemotherapy in future studies.

## 2. Susceptibility Genes in Chemotherapy-Induced Cardiotoxicity

**Figure 2:**
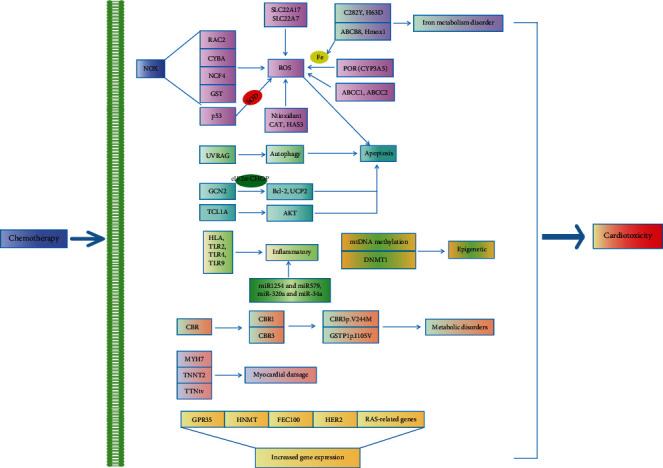
Mechanism of cardiotoxicity induced by susceptibility genes in chemotherapy. ROS: reactive oxygen species; NOX: nicotinamide adenine dinucleotide phosphate oxidase; POR: P450 oxidoreductase; GST: glutathione S-transferase; CYP3A5: cytochrome P450 family 3 subfamily A member 5; CAT: catalase; HAS3: hyaluronan synthase 3; SOD: superoxide dismutase; UVRAG: ultraviolet irradiation resistance-associated gene; GCN2: general control nonderepressible 2; eIF2*α*: eukaryotic initiation factor 2*α*; UCP2: uncoupling protein 2; Bcl-2: B-cell lymphoma-2; TCL1A: T cell leukemia/lymphoma 1A; HLA: human leukocyte antigen; TLR2: Toll-like receptor 2; TLR4: Toll-like receptor 4; TLR9: Toll-like receptor 9; Hmox1: heme oxygenase-1; CBR: carbonyl reductase; CBR1: carbonyl reductase 1; CBR3: carbonyl reductase 3; TTNtv: titin-truncating variants; DNMT1: DNA methyltransferase 1; GPR35: G protein-coupled receptor 35; HNMT: histamine n-ethyltransferase; RAS-related genes: renin-angiotensin system-related genes.

Genes positively correlated with cardiotoxicity have been found to contain alleles that change the encoding of protein expression, leading to the development of disease [17, 18]. Genetic markers that predict whether patients will develop cardiotoxicity from chemotherapy would allow for the careful monitoring of patients, the administration of cardioprotective drugs, and the early initiation of treatment after cardiotoxicity [19–21]. This review provides an overview of all the genetic variants that have been found to influence susceptibility to cardiotoxicity (Table 1 and Figure 2). The identified gene variants are discussed in view of the latest theories regarding the complex pathological mechanisms responsible for this adverse drug reaction.

### 2.1. Oxidative Stress

Chemotherapy produces reactive oxygen species (ROS) via multiple pathways, including hydroxyl radicals (-OH), superoxide radicals (O^2-^), and hydrogen peroxide (H_2_O_2_). Excessive ROS generation is the most widely theorized mechanism for mediating chemotherapy-induced cardiotoxicity [22–24]. H_2_O_2_ and O^2-^ may generate the toxic -OH and cause myocardial injury [25]. The heart is particularly vulnerable to oxidative stress because of the low levels of enzymes that neutralize these substances found in cardiac tissue [26, 27]. ROS interacts with DNA, proteins, and lipid membranes to destroy them.

Chemotherapy produces excessive free radicals by exploiting cellular oxidoreductases, including nicotinamide adenine dinucleotide phosphate (NADPH) and nicotinamide adenine dinucleotide hydrogen (NADH) dehydrogenase, resulting in cardiotoxicity [28–30]. The NADPH oxidase (NOX) multienzyme complex uses NADPH or NADH as an electron donor to promote a 1-electron reduction of oxygen. This enzyme has been studied in the endothelium and macrophages, and was recently confirmed as a possible primary source of ROS in the myocardium [31]. Genotypic variations of alpha-1 class glutathione S-transferase (GSTA1, rs3957357) and NOX p22phox (CYBA, rs4673) are predictors of event-free survival. The influence of single-nucleotide polymorphisms (SNPs) on toxicity was assessed in 658 rituximab-CHOP- (R-CHOP-) 21 courses [32]. Overall, the SNPs influencing CYBA rs4673 and GSTA1 rs3957357 may predict patient prognosis after R-CHOP-21 treatment. In addition, a variant of the NOX subunit NCF4 (rs1883112) may prevent hematological and nonhematological toxicity [33, 34]. Another study investigated genotype participants and conducted a follow-up study for the occurrence and development of HF [35]. The SNPs were selected from 82 genes potentially associated with cardiotoxicity. Among 1,697 patients, 55 had acute anthracycline-induced cardiotoxicity (ACT) and 54 had chronic ACT. This study detected 5 genes that were related to polymorphisms in NOX and doxorubicin (DOX) efflux transporters, while chronic ACT was found to be related to NCF4 (rs1883112). Additionally, acute ACT was found to be related to the p22phox subunit (rs4673) and the RAC2 subunit (rs13058338). Consistent with these results, mice with insufficient NOX activity were resistant to chronic DOX therapy [35–37].

Meanwhile, another previous study investigated 2,950 patients who had undergone hematopoietic cell transplantation (HCT) from 1988 to 2007 [38]. Genotyping was performed on 77 cases of HCT germline DNA and 178 cases of control. The results of multivariate analysis showed that the incidence of congestive heart failure (CHF) was higher in patients with pre-HCT chest radiation and with gene variants coding for the NOX subunit RAC2 (rs13058338), HFE (rs1799945), or the DOX efflux transporter ATP-binding cassette subfamily C member 2 (ABCC2, rs8187710) [35, 39]. In addition, the polymorphisms of NOX subunits and transporters ABCC1, ABCC2, and SLC28A3 were genotyped in patients with aggressive CD20 B-cell lymphoma [40, 41]. The RAC2 subunit genotypes were found to have statistical significance in the multivariate logistic regression analysis. In summary, RAC2 and CYBA genotypes appear to be related to ACT [34, 42], which demonstrates that NOX is associated with ACT.

ABCC1, also known as multidrug resistance-associated protein 1 (MRP1), is expressed in the heart and is involved in detoxifying and protecting against the toxic actions of xenoorganisms [43, 44]. One study investigated the correlation between left ventricular (LV) function and SNPs in the ABCC1 gene in children treated with anthracyclines [45]. The data of acute lymphoblastic leukemia in children were analyzed, and echocardiography and genotyping of 9 polymorphisms of the ABCC1 gene were performed. The results revealed that the combination of ABCC1 rs3743527TT and rs3743527tt-rs246221tc/TT is associated with lower LV fractional shortening (FS), suggesting that genetic variations in the ABCC1 gene may impact LV dysfunction induced by anthracycline. Moreover, the synonymous encoding variant rs7853758 in the SLC28A3 gene was significantly related to ACT [46–48]. The risk and protection variants of other genes have been described, including SLC28A1 and several kinds of ATP-binding cassette transporters (ABCB1, ABCB4, and ABCC1). The novel relevance of the Top2b (topoisomerase-IIb) SNPs was verified [49], which suggested an association between the SNPs of RAC2, NCF4, and SLC28A3, and 23 SNPs associated with ACT [50]. Another study examined the relationship between 36 candidate polymorphisms of MAP (methotrexate, adriamycin, and cisplatin) pathway genes and grade 3-4 chemotherapy toxicity [48]. Blood samples were taken from patients who had completed MAP chemotherapy. All patients were manually genotyped to identify five polymorphisms, while the remaining 31 polymorphisms were genotyped using Illumina 610-Quad microarray. The results suggested that the toxicity of methotrexate was enhanced in the MTHFR, ABCB1, and ABCC2 variants [48, 51, 52].

The P450 oxidoreductase (POR) gene encodes a flavin protein that transfers electrons from NADPH to various kinds of proteins, including the cytochrome P450 enzymes [53]. Anthracyclines and other quinone compounds are transformed by microsomes into hemiquinone radical form through an electron reduction reaction catalyzed by POR. This biological activation step stabilizes the drug's cross-linking to DNA and is thought to greatly enhance its cytotoxicity [54]. This study detected 60 gene-encoding proteins participating in drug metabolism and efflux, with the POR gene and daunorubicin (DNR) showing the strongest cardiotoxic effects in patients with acute myeloid leukemia (AML) [55]. In this cohort of patients with AML, the estimated variation in the POR gene after DNR treatment accounted for approximately 11.6% of the LVEF-decreased patients and 13.2% of the LVEF-decreased patients with a cumulative dose. In post hoc analysis, this association was driven by a linear interaction of 3 SNPs (rs2868177, rs13240755, and rs4732513) with a cumulative dose of DNR. Another study examined the relationship between cytochrome P450 family 3 subfamily A member 5 (CYP3A5) genetic polymorphism and the DNR plasma concentration in patients with AML [56]. The study included 36 children who had been recently diagnosed with acute lymphoblastic leukemia (ALL). Polymerase chain reaction- (PCR-) derived sequencing was used to detect the CYP3A5∗3 genotype, and then PCR was used to detect the mRNA expression of CYP3A5. The enzyme activity of CYP3A was detected using a midazolam probe, and the DNR concentration was determined via high-performance liquid chromatography. The expression levels of CYP3A5 mRNA in children with different genotypes were different, while the activity of the CYP3A5 enzyme in the CYP3A5∗1 allele was higher than that in the CYP3A5∗3 allele. The polymorphism of the CYP3A5∗3 gene is closely related to CYP3A enzyme activity, the mRNA expression of CYP3A5, and the DNR plasma drug concentration, and results in different adverse drug reactions [56–58].

The evidence is increasingly indicating that drug metabolizing enzymes, such as the members of the glutathione S-transferase (GST) family, have great effect for characterizing the response of patients to chemotherapeutic drugs [59, 60]. The corresponding genes, such as GSTM1, glutathione s-transferase Pi (GSTP1), and GSTT1, encode the phase II detoxifying proteins that are involved in conjugating substrates that are toxic to cancer cells, including the type of chemotherapy used in the treatment of breast cancer [61–63]. However, the key participant in the pathophysiology of CHF is the renin-angiotensin-aldosterone system (RAAS) [64]. This study determined whether polymorphisms in the RAAS and GST II detoxification enzyme families might be useful predictors of LVEF dynamics and CHF risk [65]. The association between the gene polymorphisms and cardiotoxicity development was investigated in 48 early breast cancer patients undergoing anthracycline-assisted chemotherapy. The following polymorphisms were analyzed: p.Met235Thr and p.Thr174Met in angiotensinogen (AGT), Ins/Del in angiotensin-converting enzyme (ACE), A1166C in angiotensin II type 1 receptor (AGTR1A), and p.Ile105Val in GSTP1 in c.-344t>c aldosterone synthase (CYP11B2). In addition, GSTM1 can be used as a biomarker with a higher risk of cardiotoxicity, as demonstrated previously in patient cohorts [62, 66, 67].

The cardiotoxicity of anthracyclines is thought to be caused by cardiomyocyte damage mediated by ROS, which is produced by the mitochondrial respiratory chain and the nonenzymatic iron pathways. A high oxidative metabolic rate and weak antioxidant defense make cardiomyocytes especially sensitive to free radical damage [68–70]. Catalase (CAT), GSTT1, GSTM1, and superoxide dismutase II (SOD2) play important roles in ROS metabolism. Rajić et al. demonstrated that deactivating the variants of CAT (rs1001179 and rs10836235), SOD2 (rs4880), GSTM1, and GSTT1 may increase cardiotoxicity risk [71]. This hypothesis was investigated in a long-term survival cohort of 76 children with ALL. Compared to genetic polymorphisms, cardiac injury was assessed as a property variable [72]. The results suggested a significant association between CAT (rs10836235) and cardiac damage after exposure to anthracyclines. The most important gene was electron transfer flavoprotein beta subunit (ETFB, rs79338777), which participated in mitochondrial b oxidation and adenosine triphosphate (ATP) production, and whose association was replicated in a group of independent cancer patients treated with anthracyclines [73, 74].

An additional study investigated whether targeted damage to the p53 gene could enhance the cardiotoxicity induced by DOX [75, 76] by randomly assigning wild-type (WT) mice and p53 knockout (p53 KO) mice to saline or DOX by intraperitoneal injection. The continuous imaging of animals using high-frequency two-dimensional echocardiography and the LV systolic function measurements assessed by FS indicated weight loss in the WT mice as early as 4 days and 2 weeks after DOX injection. On the contrary, LVFS remained unchanged after DOX injection in the p53 KO mice. After DOX treatment, the apoptosis of cardiomyocytes measured using TUNEL and the ligase reaction were found to increase significantly, whereas the level of glutathione and Cu/Zn SOD did not change in the p53 KO mice, but not in the WT mice. Therefore, the p53 gene in p53-mediated signaling may play an important role in the cardiotoxicity induced by DOX, and may regulate ROS induced by DOX [77].

Hyaluronan (HA) generated by hyaluronan synthase 3 (HAS3) is a common ingredient and has a positive effect on a variety of diseases [78]. Furthermore, HA is known to decrease heart damage caused by ROS in cardiovascular disease. This study examined host sensitivity to anthracycline-associated cardiomyopathy using a cardiovascular SNP array to analyze common SNPs in 2,100 genes associated with cardiovascular disease [79]. The study identified a common SNP (rs2232228) in the HAS3 gene that modifies the risk of anthracycline-induced cardiomyopathy. Compared to the GG genotype, the rs2232228 AA genotype increased the risk of cardiomyopathy by 8.9 times [38].

SLC22A17 was first identified in the brain as an orphan transporter of unknown endogenous substrates, expressed in a variety of tissues, including the heart [80]. SLC22A17 transports naturally occurring nucleotides, preferentially selects guanine analogs and several nucleoside-based drugs, and has a considerable substrate overlap with concentrated nucleoside transporters [81, 82]. This study verified novel variants related to ACT and evaluated them in a risk prediction model. Two cohorts for the treatment of childhood cancer were genotyped for 4,578 SNPs in the drug ADME (absorption, distribution, metabolism, and elimination) and toxicity genes [83]. An important association between SLC22A7 (rs4149178) and SLC22A17 (rs4982753) was found, and evidence was also found for some genes associated with ROS [84]. Two new variants in SLC22A17 and SLC22A7 were associated with cardiotoxicity induced by anthracyclines, thereby improving risk stratification in patients.

### 2.2. Autophagy

Autophagy in its normal state is essential for maintaining homeostasis [85, 86]; however, disorders of autophagy in cardiomyocytes have been linked to a variety of cardiovascular diseases [87–89]. Autophagy is associated with cardiomyopathy induced by DOX [90–95], and the ultraviolet irradiation resistance-associated gene (UVRAG), an autophagy-related protein, can adjust autophagosome formation [96], maturation [97], and autophagosomal lysosomal reformation (ALR) [98]. Studies on UVRAG-deficient mice found that the autophagy flux was impaired and autophagosomes were accumulated in the heart, suggesting that UVRAG may regulate the maturation of autophagosomes [99, 100]. An et al. evaluated the effect of UVRAG-mediated autophagy in cardiotoxicity induced by DOX [101]. The deficiency of UVRAG will aggravate the cardiotoxicity induced by DOX, which is manifested by an enhancement of cytoplasmic vacuoles, an increased collagen accumulation, increased serum levels of lactate dehydrogenase (LDH) and myocardial creatine kinase (CK), increased ROS levels, increased apoptosis, and reduced cardiac function. The autophagy flux was impaired in cardiotoxicity induced by DOX, while a deficiency of UVRAG exacerbated autophagy flux impairment in cardiotoxicity induced by DOX. In summary, these data suggest that UVRAG deficiency in part aggravates cardiotoxicity by exacerbating DOX-induced autophagy impairment.

### 2.3. Apoptosis

General control nonderepressible 2 (GCN2) is a eukaryotic initiation factor 2*α* (eIF2*α*) kinase that damages ventricular adaptation to pressure overload by influencing myocardial apoptosis [102]. After DOX treatment, systolic dysfunction, apoptosis, and ROS were found to be reduced in Gcn2^−/−^ mice. GCN2 deficiency attenuated eIF2 phosphorylation, induced its downstream targets, activated transcription factor 4 (ATF4) and C/EBP homologous protein (CHOP), and retained B-cell lymphoma-2 (Bcl-2) and mitochondrial uncoupling protein 2 (UCP2). In addition, this study found that the knockdown of GCN2 weakened DOX-induced ROS, while the overexpression of GCN2 intensified it, and reduced Bcl-2 and UCP2 through the eIF2*α*-CHOP pathway [103–105]. Furthermore, another study found that oxidative byproducts accumulated in the plasma of patients treated with DOX [106]. At the RNA level, compared with women who received chemotherapy but maintained normal EF, the 260 transcripts of women with low EF changed after chemotherapy, with a difference of >2 times. Notably, the transcription of T cell leukemia/lymphoma 1A (TCL1A) decreased by 4.8 times in women with chemotherapy-induced low EF. TCL1A, also known as an AKT helper activator, is one of the primary presurvival factors of cardiac myocytes. In addition, patients with low EFs had a twofold reduction in ABCB1 transcription encoding multidrug resistant protein 1 (MDR1), which may lead to higher cardiac drug levels [107, 108]. Hence, cancer treatment-induced cardiomyopathy may result in genetic susceptibility or decreased TCL1A levels, decreased AKT activity, and augmented sensitivity to DOX apoptosis.

### 2.4. Inflammation

Previous studies have found that individual susceptibility to low doses of DOX treatment is related to the differential expression of genes involved in the inflammatory response [109], which correlates with increasing reports on the important function of human leukocyte antigen (HLA) to the hypersensitivity of complex polymorphism to drug toxicity [110]. A study analyzing DNA from breast cancer patients treated with DOX and its role in the DOX-related cardiotoxicity risk identified 18 SNPs of 9 genes in the HLA region that may be associated with DOX cardiotoxicity [109, 111]. This result suggested that increased susceptibility to DOX-induced cardiotoxicity is associated with the dysregulation of autoimmune and inflammatory disease-related genes [111]. In addition, Mori et al. treated rats with three typical cardiotoxic compounds, namely, isoproterenol, DOX, and carbofuran, which resulted in cardiac lesions in rats [112]. This study was followed by microarray analysis and histopathological examination. Using statistical and cluster analysis, 36 probe groups were extracted from the upregulation of three cardiotoxic compounds. The analysis showed that these genes were involved in the myocardial degeneration and inflammation observed in histopathological analysis. Among the selected genes, Timp1, Spp1, Ccl7, Fhl1, and Reg3b showed a sustained upregulation of high expression levels in all three compounds at both time points [113–115].

Toll-like receptors (TLRS), including TLR4, TLR2, and TLR9, allow cardiomyocytes to respond to endogenous or exogenous stimuli, and may alter their pathophysiological response [116, 117]. One study investigated the potential role of TLR2 and TLR4 gene expression as early biomarkers of cardiomyopathy induced by DOX [118]. In this study, blood collection, RNA isolation, cDNA reverse transcription, quantitative reverse transcription PCR (qRT-PCR), and relative expression quantification were performed on samples from 25 patients with DOX-treated hematologic malignancies via qRT-PCR. The results showed that TLR4 and TLR2 expression was higher in patients with diastolic dysfunction and DOX treatment [118, 119]. In addition, DOX was found to participate in PI3K*γ* downstream signaling of TLR9, which converged to autophagy inhibition and maladaptive metabolic remodeling, ultimately leading to cardiomyocyte death and systolic dysfunction. One study treated chronic DOX in mice expressing inactive PI3K*γ* or receiving selective PI3K*γ* inhibitors [120]. Cardiac function was assessed by echocardiography, and DOX-mediated signaling was evaluated in the heart tissue and cardiomyocytes. The dual cardioprotective and anticancer effects of PI3K*γ* inhibition were evaluated in mice tumor models. The results showed that PI3K*γ* kinase dead (KD) mice exhibited preserved cardiac function after a long-term low dose of DOX therapy and were protected by DOX-induced cardiotoxicity. The effect of PI3K*γ* inhibition was found to have a causal relationship with enhanced autophagy processing in the DOX-damaged mitochondria. In terms of its mechanism, PI3K*γ* was triggered downstream of TLR9 in DOX-treated mice hearts by mitochondrial DNA released by damaged organelles and contained in the autolysosomes [121, 122].

### 2.5. MicroRNAs (miRNAs)

MicroRNAs (miRNAs) are universally expressed small noncoding RNAs, which adjust gene expression at the posttranscriptional level [123]. The importance of miRNAs in a wide range of human diseases suggests their potential as biomarkers for clinical use [124]. Numerous studies have shown that miRNA expression profiles are associated with cardiovascular diseases, including fibrosis, hypertrophy, arrhythmia, and HF, and can have powerful and unexpected effects [125–128]. One study obtained information about microRNA in cancer patients treated with DOX to determine whether these patients developed cardiac abnormalities after chemotherapy [129]. Plasma from 20 breast cancer patients who had undergone DOX treatment were analyzed using quantitative RT-PCR and qPCR. The circulating microRNA profiles of patients with cardiotoxicity induced by DOX were then compared with those without cardiotoxicity induced by DOX. The results indicated that 32 microRNAs were severely misregulated in patients with cardiac dysfunction, the analysis of which suggested that they were associated with inflammation [130, 131].

Another study determined whether specific miRNA levels were discharged into the circulation due to cardiotoxicity induced by bevacizumab [132]. After miRNA array analysis using isolated RNA, this study selected 19 candidate miRNAs from the array for a validation study of 90 controls and 88 patients with cardiotoxicity induced by bevacizumab. Compared to the control group, the circulating levels of the 5 miRNAs were significantly increased in patients with cardiotoxicity induced by bevacizumab. To verify these findings, the study compared selected miRNAs in plasma from 66 patients with acute myocardial infarction (AMI) with cardiotoxicity induced by bevacizumab. The results confirmed a specific rise in the expression of two miRNAs, miR1254 and miR579, in patients with cardiotoxicity induced by bevacizumab, with miR1254 showing the strongest association with the clinical diagnosis of bevacizumab-induced cardiotoxicity [132–134].

Furthermore, some studies have suggested that miR-320a [135] and miR-34a [134] play important roles in chemotherapy-induced cardiotoxicity. After DOX treatment, miR-320a was found to increase in the cardiomyocytes, and participated in DOX-induced cardiotoxicity due to its direct targeting of VEGF-A [135]. Therefore, the overexpression of miR-320a enhanced cardiac apoptosis and caused vessel abnormalities in the heart tissue and cardiac dysfunction in mice. miR-34a had been shown to be upregulated in the myocardium and plasma of DOX-treated rats and in the H9C2 cells of rat myocardium treated with DOX [136]. In terms of its mechanism, miR-34a contributed to DOX-induced cardiotoxicity by targeting the Sirt1/p66shc pathway [136]. It was also shown that miR-34b/c was upregulated in the myocardial cell line HL-1 treated with DOX [137]. This study showed that the itchy E3 ubiquitin protein ligase (ITCH) was a direct target of miR-34b/c, and that miR-34b/c reduced HL-1 viability, promoted NF-*κ*B expression, and increased proinflammatory cytokines through ITCH downregulation [137]. Overall, these studies demonstrated that DOX treatment is associated with miRNA signaling, which may potentially predict cardiac dysfunction in breast cancer patients [138]. Thus, these data provide a basis for future studies to identify biomarkers for cardiotoxicity induced by DOX.

### 2.6. Iron Metabolism

Hereditary hemochromatosis (HH) is an inherited iron metabolism disorder that leads to tissue damage associated with excess levels of iron. Homozygotes of the C282Y mutation are present in 52-100% of HH patients [139]. Non-cancer-related idiopathic cardiomyopathy and early pathological LV remodeling were found to be higher in patients [140] than in healthy controls [141]. This study retrospectively assessed 97 consecutive necropsies for HFE genotypes, cardiac iron, and cardiac events from patients with solid and hematologic tumors [142]. The iron concentrations in the heart and liver were tested using atomic absorption spectrometry, and the HFE gene mutations related to HH were analyzed. Haplotypes 282C/63D and 282Y/63H of HFE mutations were found to be related to higher cardiac iron deposition [143]. Other studies also confirmed a link between HH associated with the mutation frequency of the HDE gene and its association with DOX-related cardiotoxicity in children at high risk of ALL [144]. C282Y and H63D were analyzed in the peripheral blood, while serum cardiac troponin-T (cTnT) and N-terminal probrain natriuretic peptide (NT-proBNP), biomarkers for heart injury and cardiomyopathy, were measured during treatment. The results suggested that the heterozygous C282Y genotype was related with multiple increases in the concentration of cTnT. LV structure and function were evaluated by echocardiography. The results showed that LVFS and end-systolic and -diastolic posterior wall thickness were abnormal in children with both alleles. In short, DOX-induced associated cardiotoxicity is associated with C282Y HFE carriers [141, 145].

DOX-dependent cardiotoxicity is presumed to occur through ROS production and cellular iron accumulation. One study found that DOX treatment produced cardiotoxicity through preferential iron accumulation in mitochondria [146]. In cardiomyocytes, DOX became concentrated in the mitochondria and enhanced mitochondrial iron and cellular ROS levels. ABCB8 is a mitochondrial protein that promotes iron output both *in vitro* and in the heart of transgenic mice, such that its overexpression was found to reduce the content of mitochondrial iron and cellular ROS, and provided protection against DOX-induced cardiomyopathy [147, 148]. The mitochondrial levels of iron were significantly higher in patients with DOX-induced cardiomyopathy than in patients with other types of cardiomyopathy or normal heart function. These results suggested that the cardiotoxic effects of DOX were caused by an accumulation of mitochondrial iron, such that reducing the mitochondrial iron levels could prevent DOX-induced cardiomyopathy.

Ferroptosis is a new form of regulatory cell death, characterized by the iron-dependent accumulation of lipid peroxides to lethal levels, which is different from apoptosis, necrosis, and autophagy morphobically, biochemically, and genetically [149, 150]. In typical apoptotic or necrotic mice, DOX-induced cardiomyocytes exhibited characteristic ferroptotic cell death. RNA sequencing results showed that heme oxygenase-1 (Hmox1) was markedly upregulated in the DOX-treated mouse heart [151]. By administering DOX to the mice, heme degradation caused by the Nrf2-mediated upregulation of Hmox1 and cardiomyopathy caused by rapid and systematic accumulation of nonheme iron were induced, but were not observed, in Nrf2-deficient mice. Since ferroptosis is driven by damage to lipid membranes, excess free iron was found to accumulate in the mitochondria, which led to lipid peroxidation in the membrane. MitoTEMPO, a mitochondria-targeted antioxidant, can rescue DOX cardiomyopathy and supports oxidative mitochondrial damage, which is the main mechanism of heart damage caused by ferroptosis.

### 2.7. Metabolism

Carbonyl reductase (CBR) catalyzes the metabolism of anthracyclines, and SNPs in CBR affect metabolic efficiency. CBRs catalyze the reduction of anthracyclines into the cardiotoxic alcohol metabolites, especially carbonyl reductase 1 (CBR1) and carbonyl reductase 3 (CBR3), whose polymorphism affects the synthesis of these metabolites [152–154]. Blanco et al. and Reinbolt et al. investigated whether the SNPs in CBR1 (1096GA) and CBR3 (V244M) altered the risk of anthracycline-associated cardiomyopathy in cancer patients [155, 156]. They found that the CBR genotype was related to an increased risk of cardiomyopathy. Another study evaluated the relationship between changes in functional cardiac parameters after treatment with anthracyclines and the polymorphism of CBR3 and GSTP1 [157]. This study included 70 patients with normal cardiac function who received anthracyclines to assess cardiac function using gated blood pool scintigraphy and echocardiography. A TaqMan probe was used to genotype the polymorphisms of 70 patients, which were verified via DNA sequencing. In terms of the CBR3p.V244M polymorphism, the systolic and diastolic parameters from GG to AA all showed a worsening trend [158]. Meanwhile, G allele carriers with the GSTP1p.I105V polymorphism were common, and PFR was significantly reduced compared to patients with the AA genotype. Therefore, the variation of CBR3 and GSTP1 may be related to changes in short-term functional cardiac parameters after chemotherapy [159, 160].

Previous studies have also suggested that 13 of the naturally existing nonsynonymous SNPs in aldo-keto reductases (AKR) and CBR decrease the metabolic rate of anthracyclines *in vitro* [161]. This study investigated these SNPs individually and jointly for their correlation with cardiotoxicity in patients with DNR induced by AML [162]. Five of the 13 SNPs showing an *in vitro* action on anthracycline drug metabolism were tested in 185 AML patients. The results indicated the *in vitro* role of nonsynonymous SNPs in the reductase genes in the metabolism of anthracycline [163]. Another study validated the evidence of a link between SNPs and cardiotoxicity in ABCB1 in breast cancer patients treated with anthracyclines [50]. An echocardiography was used to analyze 166 breast cancer patients treated with DOX, with 19 cases of abnormal systolic function and 147 control cases. After applying the appropriate statistical correction, four high-priority SNPs were detected in the main analysis, while 23 other SNPs were screened using uncorrected secondary analysis. Two SNPs, including ABCB1 and CBR3, which are associated with cardiotoxicity, were identified as a result.

### 2.8. Sarcomere Disruption

Although anthracyclines have been successfully used to treat cancer, their use is limited by their cardiotoxic side effects [164]. There are several known risk factors for anthracycline-associated cardiomyopathy (AACM) [165]; however, the absence of these known risk factors lead to the development of AACM. One study investigated whether genetic susceptibility to dilated cardiomyopathy (DCM) is a risk factor for AACM [166]. A hospital-based and two hospital registries for cancer patients treated with systemic cancer were reviewed, with an emphasis on AACM. Mutations in genetically related cardiomyopathy in selected AACM family patients were analyzed and their presymptomatic cardiology was evaluated. The study analyzed 5 AACM families with DCM and 1 AACM family member with potential early signs of mild DCM. As a result, pathogenic MYH7 mutations were identified in the two families. Moreover, in the DCM family with AACM, mutations in MYH7 c.1633G>A and c.2863G>A were identified. Therefore, it can be hypothesized that genetic susceptibility to DCM may be a potential risk factor for AACM [166, 167].

The SNP rs1786814 on the CELF4 gene is an important cut-off for the interaction between genes and the environment [168–170]. Genome-wide association studies were used to investigate the potential mechanistic implications of verified SNPs. Multivariate analysis showed that cardiomyopathy was rare and dose independent in patients with the A allele. However, in patients exposed to anthracyclines, compared to those with the GA/AA genotype, the rs1786814 GG genotype had a 10.2-fold increased cardiomyopathy risk. The CUG-BP and ETR-3-like factor proteins control the developmental regulatory splicing of TNNT2, and this gene encodes cTnT. More than one cTnT variant may cause a transient mitotic myofilament response to calcium, resulting in a reduction in contractile force. Analysis showed that the rs1786814 GG genotype was correlated with more than one TNNT2 splicing variant. In summary, this study suggests that the CELF4 (rs1786814) polymorphism modifies the dose-dependent association between anthracyclines and cardiomyopathy, possibly through pathways involving abnormal splicing of TNNT2 variants [171–173].

Titin-truncating variants (TTNtv) are observably conspicuous in DCM, occurring in 15% of outpatients and 25% of end-stage patients [174–177], but are rarely found in childhood-onset DCM [178]. Meanwhile, this study found TTNtv in 8.1% of adults and 5.0% of children with cancer treatment-induced cardiomyopathy (CCM). Garcia-Pavia et al. studied patients from three cohorts, retrospectively enrolling patients with multiple cancers, breast cancer, and AML, and sequenced their cardiomyopathy genes, including nine prespecified genes [179]. This study compared the incidence of rare mutations between the CCM cohort and the cancer genome atlas (TCGA) participants, healthy volunteers, and reference populations with matched lineages. The prevailing CCM genotype was simulated in anthracycline-treated mice based on the genotype assessment of clinical characteristics and results. Of the nine priority genes, CCM patients had more rare protein-altered variants than their peers. TTNtv was found to be dominant, occurring in 7.5% of patients with CCM. Compared to patients without TTNtv, patients with CCM TTNtv experienced more HF, atrial fibrillation, and impaired myocardial recovery. This finding is consistent with data showing that TTNtv mice treated with anthracyclines and isolated TTNtv cardiomyocytes showed persistent systolic dysfunction, which varied from that of the wild type [179, 180].

### 2.9. Epigenetics

Since mitochondrial dysfunction can dramatically reprogram the epigenome [181, 182], cardiotoxicity may also be induced by the epigenetic changes associated with mitochondrial dysfunction. For verification, the study used rats injected with DOX or saline for 8 weeks [183]. Gene expression, global DNA methylation, and the acetylation status of proteome lysine were assessed by qPCR, ELISA, and Western blot, respectively, in saline- or DOX-treated rat cardiac tissue. This study showed that DOX treatment reduced global mtDNA methylation in the heart, which was accompanied by obvious changes in the expression of multiple functional genes. DOX disrupted the cardiac mitochondrial biogenesis, which was demonstrated by the reduced ratio of mitochondrial DNA versus genomic DNA and the decreased transcription levels of several mitochondrial genes [184]. Furthermore, the transcription of genes involved in the lipid metabolism and epigenetic regulation was also affected. Western blot analysis showed that the protein acetylation patterns in DOX-treated rat heart mitochondrial fractions were different from the control. These results indicated that the interaction between epigenetic alterations and mitochondrial dysfunction may be the main determinant of DOX-induced cardiotoxicity. In addition, Ferreira et al. investigated the correlation between nanomolar DOX concentration and epigenetic-related mitochondrial adaptation [185]. H9C2 cardiomyocytes were cultured with DOX for 24 hours and then recovered in nontoxic medium for 9 days. It was found that nanomolar DOX pretreatment led to the upregulation of mitochondrial DNA transcripts, with the decrease of DNA methyltransferase 1 (DNMT1) and the global methylation levels. This result suggested that nanomolar DOX preconditioning induction may be based on epigenetic mitochondrial adaptation.

### 2.10. Others

#### 2.10.1. HER2 (erbB-2, neu)

HER2 (erbB-2, neu) is a transmembrane protein with tyrosine kinase activity but no definite physiological ligands. Milano et al. found that HER2 gene polymorphism coding for the HER2 (Ile655Val) transmembrane domain may be a predictor of cardiac toxicity [186, 187]. A case-control study tested 11 ErbB2 single-gene SNPs that led to changes in the amino acid sequence of the HER2-neu protein related to cardiotoxicity in trastuzumab therapy [188]. Only the two ErbB2 SNPS (Ile 655 Val and Pro 1170 Ala) were discovered to be mutated by single-gene SNP analysis. The HER2/neu Pro 1170 Ala polymorphism could be used to identify an increased risk of cardiotoxicity in patients receiving trastuzumab. Another study used TaqMan allele identification to genotype the HER2 655 A>G (rs1136201) genetic variation [189]. The result showed that the polymorphism of HER2 655 A>G was significantly correlated with cardiotoxicity, and supported the role of HER2 655 A>G polymorphism as a genetic marker of cardiotoxicity in trastuzumab-induced HER2-positive breast cancer patients. Roca et al. investigated the predictive value of HER2, FCGRIIA, and FCGRIIIA gene polymorphisms on cardiotoxicity [190]. A total of 132 patients with HER2-positive breast cancer were analyzed, and the results showed that the HER2-I655V genotype was significantly associated with cardiotoxicity, whereas the FCGR2A-131 H/H genotype was markedly associated with shorter event-free survival (EFS). These results may contribute to improved efficacy and reduced toxicity, leading to the selection of HER2 blockers in adjuvant therapy. Another study examined the effects of a HER2 gene polymorphism (Ile655Val) on the pharmacodynamics of trastuzumab-induced cardiotoxicity, suggesting that the presence of the Val allele may be a risk factor for cardiotoxicity induced by trastuzumab in breast cancer patients [191, 192].

#### 2.10.2. G Protein-Coupled Receptor 35 (GPR35)

The G protein-coupled receptor 35 (GPR35) is the family of G protein-coupled receptors, a membrane protein that mediates a wide range of physiological processes [193]. The *in vitro* functional analysis of cardiomyocytes suggested that the overexpression of GPR35 decreased cell viability and promoted morphological changes [194, 195]. Ruiz-Pinto et al. studied the variation association on the Illumina HumanExome BeadChip array in 83 cancer patients treated with anthracyclines [191]. A gene-based analysis identified a novel and significant association between GPR35 and chronic ACT. This study found the greatest contribution to this association in rs12468485, where the T allele was associated with lower anthracycline doses and an increased risk of chronic ACT for more severe symptomatic cardiac presentation. Using exome array data, the results indicated that GPR35 was a novel susceptibility gene associated with the induction of ACT in cancer patients during treatment [196, 197].

#### 2.10.3. Histamine N-Ethyltransferase (HNMT)

The exact relevance between histamine n-ethyltransferase (HNMT) and cardiotoxicity is currently unknown. However, it has been proposed that antihistamines may be able to reverse multidrug resistance in breast cancer cells [198]. Recent research has shown that many SNPs play a role in ACT in children. One study investigated two adult ACT sisters who had developed ACT after administration with relatively low doses of DOX [199]. One of the sisters carried the HNMT variant genotype (rs17583889), while the other was heterozygous, suggesting that these genotypes had similar effects in ACT adults. Although further studies are needed, these gene types may play important roles for the clinical application of adriamycin liposomes.

#### 2.10.4. Renin-Angiotensin System- (RAS-) Related Genes

In the heart, variations in certain renin-angiotensin system (RAS) components are frequently observed in the conditions leading to HF progression, such as ACE and angiotensin II type 1 receptor (AT1) [200–202]. One study investigated whether the renin-angiotensin-related gene could be altered using chemotherapy and radiation in a rat model [203]. Female rats were divided into three groups: the control group, the radiation (IR) group, and the chemotherapy+radiation (TC+IR) group. Left ventricular analysis was performed five months after treatment, and changes in the mRNA levels of several RAS-related genes were assessed by RT-PCR, such as angiotensinogen, renin, ACE, AT1, and vascular endothelial growth factor (VEGF), which may be involved in ACE. Compared with the control group, only decreased levels of ACE and VEGF were observed in renin, TC+IR, and IR, while increased levels of AT1 mRNA were observed in the TC+IR group and IR groups. In summary, both chemotherapy and radiotherapy may result in significant changes to the expression of some RAS-related genes [203, 204].

#### 2.10.5. Others

A genome-wide association study (GWAS) was conducted on 3,431 patients from a randomized phase III study-adjuvant breast cancer trial (E5103) to identify the SNP genotypes associated with an increased risk of CHF after treatment with anthracyclines [205]. The study attempted to validate the drug candidates in two separate phase III adjuvant trials, E1199 and BEATRICE. When CHF was assessed by a cardiologist, 11 SNPs were found, 9 of which were independent chromosomal regions associated with increased risk. A study of the two most important SNPs in E1199 showed that the SNP rs28714259 was associated with an increased risk of CHF at a critical level. Subsequently, rs28714259 was tested in BEATRICE and was found to be significantly correlated with LVEF reduction. Therefore, the SNP rs28714259 represents a validated SNP associated with anthracycline-induced CHF in breast cancer clinical trials [205, 206].

A susceptibility to the chemotherapeutic drug-induced prolongation of QT interval is thought to be associated with SNPs or genetic mutations, some of which are present in the potassium channel gene [207]. Using electrocardiograms, the QTc intervals and arrhythmia characteristics were assessed in early breast cancer patients undergoing FEC100 chemotherapy. In the treated patients, a total of 131 ECG records were obtained, and the QTc interval was measured in 127 records. After each treatment, a marked trend in QTc interval prolongation was observed, lasting for four chemotherapy cycles. In the first to the fourth chemotherapy cycle, the median length of QTc interval prolongation was 13, 11, 18, and 14 ms, respectively. In the first and fourth weeks before and after treatment, the QTc intervals were significantly different, and a supraventricular premature beat was found in 3 of the 131 cycles in 2 of the 34 patients. Therefore, this study confirmed that FEC100 is associated with significantly longer QTc intervals in early breast cancer patients [208].

CHF: congestive heart failure; LVEF: left ventricular ejection fraction; SF: shortening fraction; DLBCL: diffuse large B-cell lymphoma; AML: acute myeloid leukemia; ALL: acute lymphoblastic leukemia; OSC: osteosarcoma; NADPH: nicotinamide adenine dinucleotide phosphate; ROS: reactive oxygen species; NOX: nicotinamide adenine dinucleotide phosphate oxidase; POR: P450 oxidoreductase; GST: glutathione S-transferase; CYP3A5: cytochrome P450 family 3 subfamily A member 5; CAT: catalase; HAS3: hyaluronan synthase 3; SOD: superoxide dismutase; UVRAG: ultraviolet irradiation resistance-associated gene; GCN2: general control nonderepressible 2; eIF2*α*: eukaryotic initiation factor 2*α*; UCP2: uncoupling protein 2; Bcl-2: B-cell lymphoma-2; TCL1A: T cell leukemia/lymphoma 1A; HLA: human leukocyte antigen; TLR2: Toll-like receptor 2; TLR4: Toll-like receptor 4; TLR9: Toll-like receptor 9; Hmox1: heme oxygenase-1; CBR: carbonyl reductase; CBR1: carbonyl reductase 1; CBR3: carbonyl reductase 3; TTNtv: titin-truncating variants; GPR35: G protein-coupled receptor 35; HNMT: histamine n-ethyltransferase; RAS-related genes: renin-angiotensin system-related genes.

## 3. Protective Genes in Cancer Treatment-Induced Cardiotoxicity

**Figure 3:**
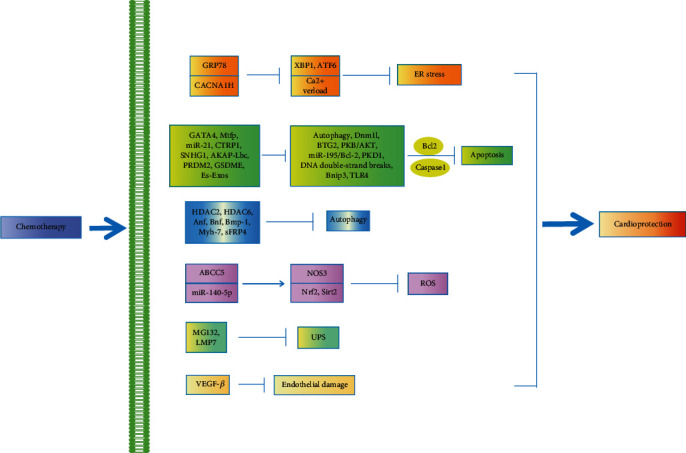
The mechanism by which genes protect against cardiotoxicity in chemotherapy. ROS: reactive oxygen species; ER stress: endoplasmic reticulum stress; ATF6: transcription factor 6; XBP1: X-box binding protein 1; GRP78 glucose regulatory protein; Mtfp1: mitochondrial fission protein 1; Dnm1l: dynamin 1-like; BTG2: B-cell translocation gene 2; CTRP1: C1q/TNF-related protein 1; PKB/AKT: protein kinase B phosphorylation; Bcl-2: B-cell lymphoma-2; AKAP: A-kinase anchoring protein; SNHG1: small nucleolar RNA host gene 1; PRDM2: PR domain-containing 2 with ZNF domain; GSDME: gasdermin D; Bnip3: Bcl-2/adenovirus E1B 19 kDa interaction protein 3; ES-Exos: embryonic stem cell-derived exosomes; TLR4: Toll-like receptor 4; UPS: ubiquitin-proteasome system; HDAC2: histone deacetylase; VEGF-*β*: vascular endothelial growth factor-*β*.

Genes are known to play important roles in various human cancers, as well as in the pathogenesis of heart development and cardiovascular disease, due to their involvement in adjusting heart function, cardiac hypertrophy, and HF [209]. The following provides a summary of various cardiac protective mechanisms and insights into the development of new drugs and personalized therapies to decrease, or even eliminate, the toxic effects of chemotherapy on the heart (Table 2 and Figure 3).

### 3.1. Oxidative Stress

Anthracycline-induced cardiotoxicity has been associated with polymorphisms in genes encoding for NOX complex subunits, namely, ABCC1 and ABCC2, among survivors of various cancers [35, 38, 45]. Krajinovic et al. analyzed 251 children with ALL using echocardiography to determine the impact of the metabolic and functional pathway polymorphism of DOX on cardiotoxicity [210]. The results of association analysis indicated a regulatory role of the variants A-1629 T (an ATP-binding cassette transporter) and G894T (the NOS3 endothelial nitric oxide synthase gene). The ABCC5 tt-1629 genotype had an average reduction in EF and SF of 8–12%, while the NOS3 TT894 genotype exerted a protective role on EF and FS in the patients [210, 211], especially in those who were not administered dexrazoxane.

Another study investigated the mechanisms and targets for DOX-induced cardiotoxicity [212]. Both *in vitro* models of cells and *in vivo* models of mice were established, the results of which indicated that DOX could significantly reduce the activity of H9C2 cells, increase the levels of LDH and CK, and induce histopathological and electrocardiac changes in mice, thereby inducing myocardial oxidative damage. An mRNA microarray assay was used to select miR-140-5p as the target miRNA responsible for a significant increase in DOX-induced cardiotoxicity. A double-luciferase reporter gene assay suggested that miR-140-5p was able to directly target Nrf2 and Sirt2, thereby increasing DOX-induced oxidative damage to the myocardium. Furthermore, the intracellular ROS levels were found to prominently increase or decrease after miR-140-5p mimic or inhibitor transfection, with changes in the expression levels of Nrf2 and Sirt2 [213–216]. In addition, DOX-induced oxidative damage to the myocardium was found to be alleviated in mice treated with a miR-140-5p antagomir. Therefore, miR-140-5p/Sirt2 and miR-140-5p/Nrf2 may become new targets for the treatment of DOX-induced cardiotoxicity.

### 3.2. Endoplasmic Reticulum (ER) Stress

It has been shown that DOX causes endoplasmic reticulum (ER) dilation in both human and mouse hearts [217, 218], suggesting that ER dysfunction is related to DOX-induced cardiotoxicity, and that the inhibition of ER stress is a feasible method to improve DOX-induced cardiotoxicity [219]. One study found that DOX caused the ER in the hearts of mice to expand, suggesting that DOX may affect ER function. DOX activated the ER transmembrane stress sensor in cultured cardiomyocytes and mouse hearts and activated transcription factor 6 (ATF6) [220]. However, DOX inhibited the expression of ATF6 downstream genes, including the X-box binding protein 1 (XBP1). Reduced levels of XBP1 resulted in an inability to induce the expression of ER chaperone glucose regulatory protein (GRP) 78, which plays a major role in the adaptive response to ER stress. Moreover, DOX activated caspase-12, an apoptotic molecule located in the ER membrane, resulting in cardiac dysfunction. In brief, DOX can activate the apoptosis response caused by ER stress, further increasing ER stress in the mouse heart. However, the overexpression of heart-specific GRP78 or the administration of the chemical ER partner alleviates the cardiac dysfunction caused by DOX.

CACNA1H was found to be related to DOX-induced cardiac toxicity, while the CACNA1H-specific inhibitor ABT-639 significantly reduced DOX-induced cardiac damage and dysfunction, and relieved ER stress and the apoptosis of cardiac myocytes [221, 222]. One study assessed DOX-induced heart damage and changes in CACNA1H expression, and investigated the effects of ER stress and apoptosis on DOX-induced heart damage in mice [222]. To determine the effect of CACNA1H in this process, this study assessed the DOX-induced changes in heart injury and ER stress after treatment with a CACNA1H-specific inhibitor, ABT-639. Lastly, the ER stress inhibitor UR906 was used to determine the effect of ER stress on DOX-induced cardiac toxicity in H9C2 cells. The results showed that DOX treatment resulted in cardiac injury, decreased cardiac function, increased myocardial cell apoptosis, and a significant increase in CACNA1H expression in the heart tissue. The CACNA1H inhibitor ABT-639 was found to partially protect cardiac function and reduce apoptosis in mice [223]. These results suggest that CACNA1H may reduce DOX-induced cardiotoxicity by decreasing the severity of ER stress, since ABT-639 significantly altered the expression of ER stress-related proteins, including PERK, P-PERK, ATF6, CHOP, ATF4, and GRP78. Therefore, the inhibition of CACNA1H may significantly reduce DOX-induced ER stress, cardiac toxicity, and apoptosis.

### 3.3. Apoptosis

Apoptosis plays an important role in cardiovascular disease. It is associated with the loss of cardiomyocytes in several kinds of heart diseases, including myocardial infarction, myocardial hypertrophy, HF, and cardiotoxicity [224–226]. Recent studies have shown that the inhibition of cardiomyocyte apoptosis can significantly reduce DOX-induced cardiac dysfunction [227–230]. Therefore, the discovery of novel genes that alleviate the apoptosis of cardiomyocytes is essential for the treatment of DOX-induced cardiotoxicity. Currently, a new mitochondrial inner membrane protein, mitochondrial fission protein 1 (Mtfp1), has been authenticated [231] and is considered to be indispensable for maintaining mitochondrial membrane integrality; it has, therefore, been associated with mitochondrial fission regulation [232]. One study reported on the role of Mtfp1 in mitochondrial division and on the induction of apoptosis in DOX-induced cardiotoxicity [233]. The knockdown of Mtfp1 can prevent mitochondrial fission in cardiomyocytes, subsequently decreasing DOX-induced apoptosis by preventing the accumulation of mitochondrial-type dynamin 1-like (Dnm1l). Conversely, when Mtfp1 is overexpressed, DOX can lead to large amounts of cardiomyocytes undergoing mitochondrial apoptosis. These results indicate that the knockdown of Mtfp1 can minimize myocardial cell loss in DOX-induced cardiotoxicity. Therefore, Mtfp1 expression regulation is a potential new treatment for cardiotoxicity induced by chemotherapy [233, 234].

The transcription factor GATA4 has been shown to influence the expression of various cardiac-related genes [235, 236]. Previous studies have shown that DOX could downregulate GATA4 transcription in myocardial cells [237, 238]. The GATA4 level protection by the 1-adrenergic agonist phenylephrine or GATA4 overexpression by the adenovirus-mediated gene transfer protected myocardial apoptosis induced by DOX [237–239]. The protective effect of GATA4 against DOX-induced cardiotoxicity is mediated at least in part by its ability to upregulate the expression of the Bcl-2 gene [240], which is a survival factor that inhibits apoptosis and autophagy. Kobayashi et al. investigated the ability of GATA4 to suppress autophagy and act as the underlying mechanism of protection against DOX-induced toxicity in cardiomyocytes [94]. DOX treatment decreased the GATA4 protein levels, leaving cardiomyocytes vulnerable to DOX-induced toxicity. Indeed, autophagy activated by GATA4 gene silencing was found to increase the toxicity of DOX, while the overexpression of GATA4 restrained the autophagy induced by DOX, thereby decreasing cardiomyocyte apoptosis. This mechanism indicates that GATA4 may upregulate Bcl-2 gene expression and inhibit the activation of autophagy-related genes induced by DOX, thus the antiapoptosis and antiautophagy roles of GATA4. These findings suggest that the activation of autophagy mediated DOX-induced cardiotoxicity, while the preservation of GATA4 inhibited autophagy by regulating the Bcl-2 and autophagy-related gene expression, thereby suppressing cardiotoxicity induced by DOX [94, 241, 242].

mRNA-21 (miR-21) plays an important role in adjusting apoptosis [243]. Although miR-21 is involved in cardiovascular disease, little is known about its biological function in response to cardiotoxicity induced by DOX. One study reported on the effects of DOX on cardiac function and miR-21 expression in mouse heart tissue and H9C2 cardiac myocytes [244]. The results suggested that the cardiac function of mice with chronic DOX injury was worse than that of mice with acute DOX injury; DOX treatment prominently enhanced the expression of miR-21 in mice cardiac tissues and H9C2 cardiomyocytes. The overexpression of miR-21 weakened apoptosis in cardiomyocytes induced by DOX and decreased the levels of miR-21 expression attenuated by the DOX-induced apoptosis of cardiomyocytes. The results of functional gain and loss experiments suggested that the B-cell translocation gene 2 (BTG2) was a target of miR-21, with BTG2 expression being prominently reduced in DOX-treated cardiomyocytes. In this study, miR-21 was found to protect mice myocardial and H9C2 cells from cardiotoxicity induced by DOX by targeting BTG [245, 246].

C1q/TNF-related protein 1 (CTRP1) is a highly conserved family of proteins [247] expressed in the heart [248, 249]. Chen et al. studied the expression of CTRP1 in the heart using an *in vivo* gene delivery system [250]. Two weeks after the gene was delivered, an intraperitoneal injection of DOX was administered to the mice to induce cardiac injury. In the DOX-treated mice, the levels of CTRP1 were reduced. The overexpression of CTRP1 then decreased cardiac troponin I, recovered cardiac function, and weakened cardiac cell apoptosis. CTRP1 expression also ameliorated cell viability and decreased the release of LDH. In contrast, DOX led to a reduction in protein kinase B phosphorylation (PKB/AKT) [251], but this was recovered by CTRP1 overexpression. The inhibition of AKT can counteract the inhibitory roles of CTRP1 on myocardial cell apoptosis [252]. In AKT-deficient mice, CTRP1 lost its ability to provide protection against cardiac damage caused by DOX. However, transfusion with recombinant CTRP1 could reverse preestablished cardiac damage caused by DOX therapy. Overall, CTRP1 provided protection against cardiotoxicity induced by DOX by activating the AKT signal pathway [250, 253]. Therefore, CTRP1 has therapeutic potential against cardiotoxicity induced by DOX.

A-kinase anchoring proteins (AKAPs) have been proposed to coordinate and synchronize the activity of a variety of signal transducers to regulate key cellular processes in the heart [254, 255]. AKAP-Lbc is a protein primarily expressed in the cardiac tissue that coordinates the activation of the hypertrophic transduction pathway downstream of *α*1-Ars [256–258]. In *in vivo* experiments, AKAP-Lbc has been shown to promote compensatory hypertrophy and cardiomyocyte protection in stress-overloaded hearts [259–261]. The stimulation of myocardial cells by the *α*1-adrenergic receptor (AR) agonist phenylephrine (PE) was found to prominently inhibit DOX-induced apoptosis [262]. Importantly, this result suggests that AKAP-Lbc is crucial for sending protection signals downstream of *α*1-Ars [263]. This study also found that the inhibition of AKAP-Lbc expression in the ventricular myocytes infected with lentivirus RNA may reduce PE's ability to reduce DOX-induced apoptosis [238]. AKAP-Lbc-mediated cardiomyocytes activate the expression of antiapoptotic protein Bcl-2 and suppress the transport of proapoptotic protein Bax to the mitochondria [239, 240]. In summary, AKAP-Lbc can provide cardiomyocytes with protection against DOX-induced toxicity.

Long noncoding RNA (lncRNA), a group of RNA molecules with lengths greater than 200 nucleotides, has limited protein-coding potential and has recently been identified as a key factor in many diseases, including cardiovascular disease [264]. lncRNA small nucleolar RNA host gene 1 (SNHG1) on human chromosome 11 has been found to be abnormally expressed in a variety of human cancers [265]. Chen et al. investigated whether DOX toxicity in AC16 cardiomyocytes *in vitro* can be adjusted by lncRNA SNHG1, with the aim of identifying potential mechanisms [266]. This study found that DOX treatment resulted in severe damage in AC16 cells by reducing cell viability and increasing cell apoptosis, while the overexpression of SNHG1 reduced apoptosis in DOX-treated AC16 cells. In addition, this study found that SNHG1 could counteract the inhibitory role of miR-195 on Bcl-2, while miR-195 restoration blocked the beneficial action of SNHG1 against DOX toxicity in AC16 cells [267]. In short, this study provided convincing evidence that SNHG1 partially protects cardiomyocytes from DOX-induced toxicity by modulating the miR-195/Bcl-2 axis [266, 267].

PR domain-containing 2 with ZNF domain (PRDM2) is crucial for the BRCA1-dependent repair of DNA double-strand breaks [268]. Damage to this mechanism increases DOX cardiotoxicity in mice [269]. In addition, PRDM2 is a heme oxygenase-1 transcriptional regulator [270], which, in addition to preventing oxidative stress [271, 272], has also been shown to promote the repair of DOX-induced DNA double-strand breaks [273] and decrease cardiomyocyte apoptosis [274]. One study examined the genetic factors that influence changes in cardiac LV function following chemotherapy with anthracyclines [275]. GWAS was conducted in this study which identified LV function changes in 385 cases of anthracyclines using BioVU after exposure to anthracyclines. The DNA samples were subsequently linked to an unidentified electronic medical record data. In a prospective clinical trial, 181 patients exposed to anthracyclines were independently replicated for variants. This study used path analysis to evaluate the combined roles of various kinds of genetic variations. These results were among the 11 candidate genes found in GWAS and located in SNP rs7542939 near PRDM2. Pathways associated with cell metabolism, DNA repair, and cardiac remodeling were identified. Therefore, using genome-wide associations, this study confirmed a susceptibility site near PRDM2 [275, 276].

Pyroptosis is a novel form of programmed cell death characterized by the swelling of cells, the blowing of large bubbles in plasma, and cytolysis, which results in the release of the cell contents and proinflammatory molecules [277, 278]. A study investigated the role of gasdermin D- (GSDME-) mediated pyroptosis in DOX-induced cardiac injury to assess the effect of BH3 protein Bcl-2/adenovirus E1B 19 kDa interaction protein 3 (Bnip3) in regulating of DOX-induced pyroptosis [279]. *In vitro* and *in vivo* cardiotoxicity models induced by DOX were established by DOX treatment. Cell transfection was used to regulate the expression of GSDME, caspase-3, and Bnip3. The release of LDH was determined using the LDH-cytotoxicity assay. Western blotting was used to measure protein level expression, flow cytometry analysis was used to determine cell death, echocardiography was used to detect heart function, and HE staining was used to observe the pathological features of the cardiac tissue. The results showed that GSDME-mediated pyroptosis was associated with DOX-induced cardiotoxicity *in vivo*. Furthermore, DOX induced the activation of caspase-3 and ultimately activated GSDME-dependent pyroptosis, which was inhibited by the silencing or inhibition of caspase-3. Other studies have shown that GSDME inhibition can inhibit the DOX-induced pyroptosis of cardiomyocytes *in vitro*. Lastly, DOX increased the expression of Bnip3, where Bnip3 silencing inhibited DOX-induced myocardial apoptosis [280, 281]. As such, this study revealed a novel pathway, the Bnip3-caspase-3-GSDME pathway, by which myocardial pyroptosis is regulated after DOX therapy.

Another study investigated whether embryonic stem cell-derived exosomes (ES-Exos) in DOX-induced cardiotoxicity attenuated inflammation-induced pyroptosis, inflammatory cell signal transduction, proinflammatory M1 macrophages, and poor cardiac remodeling [282]. To this end, the study transplanted ES-Exos and compared them with ES cells (ESCs) to detect pyroptosis, inflammation, cell signaling, adverse cardiac remodeling, and their effects on DOX-induced cardiac dysfunction. The results showed that DOX treatment significantly increased the expression of inflammasome markers (TLR4 and NLRP3), pyroptotic markers (caspase-1, IL1-*β*, and IL-18), cellular signaling proteins (MyD88, p-P38, and p-JNK), proinflammatory M1 macrophages, and TNF-*α* cytokines. ES-Exos or ESCs inhibited this increased expression of pyroptosis, inflammation, and cell signaling proteins. In addition, ES-Exos or ESCs increased M2 macrophages and anti-inflammatory cytokine IL-10, significantly inhibited cytoplasmic vacuoles and hypertrophy, and improved cardiac function [283, 284].

### 3.4. Proteasome Activity

DOX enhanced ubiquitin-proteasome system- (UPS-) mediated proteolysis in the heart, indicating that UPS hyperfunction may be an important mechanism of DOX-induced acute cardiotoxicity [285–287]. The O-linked attachment of monosaccharide-N-acetylglucosamine (O-GlcNAc) is a highly dynamic and ubiquitous protein modification [288]. Protein O-GlcNAcylation has rapidly become a key regulator of several important biological processes, including proteasomal degradation and apoptosis. However, proteasome inhibition has been found to be very effective in inhibiting cell proliferation in the treatment of cancer and for preventing restenosis [289]. These findings also suggest that the use of DOX with antitumor proteasome inhibitors may reduce the toxicity of DOX. Moreover, the overexpression of immunoproteasome-catalyzed subunits was found to markedly attenuate DOX-induced myocyte apoptosis and other UPS gene expression [290], while its knockdown significantly increased DOX-induced myocyte apoptosis [291].

UPS has been reported to be involved in Cx43 degradation [292]. The proteasome inhibitor MG132 has been found to suppress the internalization and degradation of Cx43 [293, 294]. This study investigated the roles of the MG132 proteasome inhibitor on Cx43, Zo-1, and 20S proteasome, and ubiquitin expression levels in adriamycin-induced HF rats [295]. MG132 was found to reduce adriamycin-induced injury in HF. Moreover, MG132 suppressed the expression of 20S proteasome and ubiquitin, while upregulating Cx43 and ZO-1. These findings indicate that inhibiting UPS upregulates Cx43 expression and suggest that proteasome inhibitors may be used against Cx43 degradation, thus preventing CX43-mediated arrhythmia in HF.

In another study, the role of UPS as a key monitoring pathway for maintaining cell viability and counteracting the toxicity of DOX treatment was also reported [296]. In addition to DOX treatment, the inhibition of proteasome activity is another reasonable strategy for the treatment of multiple myeloma (MM). As such, the mechanism by which small molecular compounds with clinically relevant proteasome subunit specificity affect DOX cytotoxicity was investigated. The activity of the b5 standard proteasome subunits was found to be critical in limiting off-target cytotoxicity in primary cardiomyocytes during DOX therapy. LMP7 inhibition in primary cardiomyocytes or the genetic ablation of LMP7 in cardiac tissue did not affect the development of DOX cardiotoxicity. These results suggest that immunoproteasome-specific inhibitors with known antitumor activity against MM cells may be beneficial in reducing cardiomyocyte death, compared with the compound carfilzomib [297], which targeted both the b5 standard proteasome and the LMP7 immunoproteasome subunit.

### 3.5. Histone Deacetylase (HDAC) Inhibitors

Histone deacetylases (HDACs) are widely expressed enzymes that can catalyze the removal of acetyl groups from histones, resulting in reduced DNA accessibility and gene silencing [298]. Although the exact mechanism of HDAC inhibitors in chemotherapy-induced cardiotoxicity is unclear, HDAC inhibitors are known to have a variety of effects [299–301]. Song et al. showed that HDAC6 was upregulated in DOX-treated cardiomyocytes *in vitro* and in an *in vivo* mice model, resulting in the deacetylation of *α*-tubulin [302]. Therefore, the genetic or pharmacological inhibition action of HDAC6 in mice has a cardioprotective effect on DOX by restoring the autophagic flux. In another study, Hanf et al. proved that DOX treatment affected the expression level of HDAC (SIRT1 and HDAC2) [303]. Nevertheless, pterostilbene, a natural analog of resveratrol and an antioxidant, was found to reduce cardiotoxicity induced by DOX both *in vitro* and *in vivo* [304]. This effect was attributed to the increased deacetylation activity of SIRT1, indicating its cardioprotective effect on DOX. In summary, HDAC inhibitors have a cardioprotective effect on DOX [305]. In Piotrowska et al.'s study, it was found that DOX, in a generally considered “safe” dose, caused adverse myocardial changes as soon as 2 weeks after continuous infusion in a mature chronic DOX infusion mouse model [306, 307]. The study also found that the low doses of DOX led to specific changes in several of the HDAC transcription profiles, which are epigenetic regulators of heart remodeling. These results indicated a potential cardioprotective therapy by modulating HDAC (Hdac2, Hdac4, Hdac6, and Hdac7) expression or activity during DOX treatment. Another study used various combinations of DNA methyltransferase and HDAC inhibitors, including DC301, DC302, and DC303 [308]. Induced by DC301 and DC302, Wharton's jelly mesenchymal stem cells (WJMSCs) differentiated into myocardial structures with Wnt antagonists, sFRP3 and sFRP4, and Dickkopf 1 (Dkk1) and Dkk3 upregulated. Cardiac progenitor cells were injected in vivo in a DOX-induced cardiotoxic mouse model. Bisulfite sequencing was used to examine the promoter methylation status of the cardiac transcription factor Nkx2.5 and the Wnt antagonist secreted frizzled-related protein 4 (sFRP4) after cardiac differentiation and revealed that sFRP4 was activated by promoter CpG island demethylation during cardiogenesis. The MSC-derived cardiac progenitors not only successfully transplanted to the site of DOX-induced cardiac injury in mice but also formed functional cardiomyocytes and recovered cardiac function [309–311]. These studies revealed the connection between Wnt inhibition and epigenetic modification to activate cardiac differentiation, which could strengthen the efficacy of stem cells in the treatment of cardiac injury.

### 3.6. Others

Vascular endothelial growth factor-*β* (VEGF-*β*), which promotes coronary angiogenesis and physiological cardiac hypertrophy, has potential for protection against DOX-induced cardiotoxicity [312]. In one study, doses at simulated clinical concentrations were administered to adenoviral vectors or control vectors expressing VEGF-*β* in normal mice 1 week prior to DOX treatment [313]. VEGF-*β* treatment suppressed DOX-induced heart atrophy, protected the sparse capillaries in the heart, and ameliorated the endothelial function of DOX-treated mice. VEGF-*β* also increased the volume of the LV without compromising cardiac function and decreased the expression of genes related to cardiovascular disease [314–316]. Importantly, VEGF-*β* did not affect tumor growth. As such, the inhibition of DOX-induced endothelial injury and the prevention of chemotherapy-related cardiotoxicity provide new therapeutic directions.

ALL: acute lymphoblastic leukemia; LV: left ventricular; LVFS: left ventricular fractional shortening; LVEF: left ventricular ejection fraction; HF: heart failure; UPP: ubiquitin-proteasome pathway; UPS: ubiquitin-proteasome system; LDH: lactic dehydrogenase; CK-MB: creatine kinase-MB; ROS: reactive oxygen species; ER: endoplasmic reticulum; ATF6: transcription factor 6; XBP1: X-box binding protein 1; GRP78 glucose regulatory protein; Mtfp1: mitochondrial fission protein 1; Dnm1l: dynamin 1-like; BTG2: B-cell translocation gene 2; CTRP1: C1q/TNF-related protein 1; PKB/AKT: protein kinase B phosphorylation; Bcl-2: B-cell lymphoma-2; AKAP: A-kinase anchoring protein; SNHG1: small nucleolar RNA host gene 1; PRDM2: PR domain-containing 2 with ZNF domain; GSDME: gasdermin D; Bnip3: Bcl-2/adenovirus E1B 19 kDa interaction protein 3; ES-Exos: embryonic stem cell-derived exosomes; TLR4: Toll-like receptor 4; HDAC: histone deacetylase; HDAC2: histone deacetylase 2; sFRP4: secreted frizzled-related protein 4; VEGF-*β*: vascular endothelial growth factor-*β*.

## 4. Discussion

This review provides an integrated overview of all the genetic variations that have been found to affect susceptibility to cardiotoxicity induced by chemotherapy. Genetics provides an insight into the development of toxicity associated with these cancer treatments, and by identifying the functional genetic variants related to these toxicities, we can improve our understanding of the potential mechanisms and pathways, thus paving the way for the development of novel therapies for these toxicities [317]. In addition, genetic markers with underlying predictive power could be used to identify patients who would benefit from careful monitoring and the prescription of cardioprotective drugs. Once chemotherapy-induced cardiotoxicity occurs, the use of appropriate therapeutic measures can alleviate this toxicity [18, 318, 319]. Meanwhile, clinicians can select specific treatments for patients according to the genotype studied and compare the differences in drug efficacy, toxicity, and side effects among patients with different genotypes [320]. Gene polymorphisms are closely related to individual differences in the effect of drugs. The research results are applied to rational drug use, thereby providing guidance for clinical drug therapy of tumors.

The majority of genes studied were related to biochemical pathways of chemotherapy-induced cardiotoxicity. For these genes, animal and mechanism studies have shown that their alleles changed the expression or activity levels of the encoded protein, thereby promoting the occurrence and development of disease. Cardiac toxicity results from oxidative stress, autophagy, apoptosis, inflammation, DNA damage, metabolism, and sarcoplasmic reticulum, among others. To date, several potential cellular and molecular mechanisms involving several genes for cardiotoxicity have been identified. Accordingly, the main susceptibility genes related to cardiotoxicity after chemotherapy are CYBA, GSTA1, NCF4, RAC2, ABCC1, ABCC2, CAT, UVRAG, GCN2, TCL1A, TLRS, C282Y, Hmox1, CBRs, MYH7, TNNT2, and TTNtv.

ROS is considered the primary mediator of chemotherapy-induced cardiotoxicity. Mitochondria are abundant in cardiomyocytes and are the main source of ROS. Changes in gene expression (CYBA, GSTA1, NCF4, RAC2, ABCC1, ABCC2, and CAT) lead to mitochondrial dysfunction, which results in increased ROS production and, ultimately, muscle cell damage. The turnover of damaged mitochondria via autophagy is essential to maintain the structure and function of cardiomyocytes [321], and UVRAG deficiency exacerbates DOX-induced cardiotoxicity. Moreover, a decreased ratio of Bcl-2/Bax can lead to the formation of pores in the mitochondria and the activation of the apoptotic pathway [322, 323]. GCN2 deficiency confers resistance to DOX-induced cardiomyocyte apoptosis by increasing the ratio of Bcl-2 and Bax. Moreover, an accumulation of iron (C282Y and Hmox1) in the mitochondria has recently been shown to cause chemotherapy cardiotoxicity, primarily by promoting ROS generation. Meanwhile, DOX-induced cardiac injury was found to be morphologically characterized by inflammation [324]. The genes TCL1A, TLR4, TLR2, and TLR9 appear to be strongly related with the inflammation and repair processes that occur following myocardial injury.

This study has some limitations which deserve discussion. Firstly, we found a total of 64 articles associated with chemotherapy-induced cardiotoxicity. Most of the studies were single case and animal studies and there were inconsistencies in the results reported between the studies. Secondly, the majority of the included studies had a small sample size. To ensure that the research results more effectively influence the development of personalized medicines, future studies should use large populations. Finally, the participants had different backgrounds. Multicenter research on patients from other regions, particularly Asia, Australia, Africa, Oceania, and South America, should be performed. Furthermore, an objective definition of cardiotoxicity and the frequency of events for each genotype should be considered. We also selectively discussed the role of genes included in the literature. It should be noted that the genes discussed in this review do not mean that they are superior to the other genes identified. Therefore, high-quality studies are needed to determine the susceptibility genes in chemotherapy-induced cardiotoxicity, thus providing guidance for clinical drug therapy of tumors.

## 5. Conclusion

In recent times, with improved treatment regimens, cancer patients have a better chance of survival. Unfortunately, they are at risk of developing long-term cardiotoxicity because of their anticancer therapies. However, there is a serious lack of reliable and sensitive biomarkers for the clinical evaluation of chemotherapy-induced cardiotoxicity. Based on genetic analyses, the combination of chemotherapy-induced cardiotoxicity and treatment targeting molecular targets of specific genes may prevent or mitigate the cardiotoxicity induced by chemotherapy in patients. In the context of inevitable cardiotoxicity, the effective and safe treatment of different types of cancer is important and deserves further study. This review reveals a number of potential therapeutic targets and provides a viable hypothesis for the development of new gene-targeted drugs for the treatment of chemotherapy-induced cardiotoxicity. But more high-quality studies are needed to determine the susceptibility genes in chemotherapy-induced cardiotoxicity, thus providing guidance for clinical drug therapy of tumors.

## Figures and Tables

**Table 1:** Susceptibility genes in chemotherapy-induced cardiotoxicity.

Study	Drug used	Type of cancer examined	Gene	SNP ID/location of pathogenic mutation	Targets	Cardiac toxicity	References
Rossi et al. (2009)	Doxorubicin	Large B-cell lymphoma	CYBA GSTA1 NCF4	rs4673 rs3957357 rs1883112	NAD(P)H oxidase, p40phox	EF decreased, echocardiography abnormalities, electrocardiogram abnormalities	[32]
Wojnowski et al. (2005)	Doxorubicin	Non-Hodgkin's lymphoma	NCF4 His72Tyr 7508T3A Gly671Val Val1188Glu Cys1515Tyr	rs1883112 rs4673 rs1305833 rs8187694 rs8187710	NAD(P)H oxidase, p22phox	Arrhythmia, myocarditis-pericarditis, acute HF	[35]
Armenian et al. (2013)	Anthracyclines	Hematopoietic cell transplantation	RAC2 HFE	rs8187710 rs13058338 rs1799945	NAD(P)H oxidase	CHF, depressed EF or SF	[38]
Reichwagen et al. (2015)	Anthracyclines	CD20+ B-cell lymphomas	RAC2 CYBA	rs13058338 rs4673	NADPH oxidase	Arrhythmia, reduced EF, ischemia	[40]
Sági et al. (2018)	Anthracyclines	ALL, OSC	CYP3A5 SLC28A3 ABCC2 NQO1 SLC22A6	rs4646450 rs7853758 rs3740066 rs1043470 rs6591722	ROS	LV function, SF, EF	[41]
Semsei et al. (2012)	Anthracyclines	ALL	ABCC1	rs3743527	ROS	LV dysfunction, reduced LVFS	[45]
Visscher et al. (2013)	Anthracyclines	Childhood cancer	UGT1A6 SLC28A3	rs17863783 rs7853758 rs885004	No report	SF < 26%	[46]
Visscher et al. (2012)	Anthracyclines	Childhood cancer	SLC28A3	rs7853758	No report	CHF, SF < 26%	[47]
Windsor et al. (2012)	Methotrexate	Malignant bone tumor	ABCC2 GSTP1	No report	ROS	Cardiac dysfunction, EF decreased	[48]
Hertz et al. (2016)	Doxorubicin	Breast cancer	ABCB1 CBR3	No report	Metabolism	EF < 55%	[50]
Lubieniecka et al. (2013)	Anthracyclines	AML	POR	rs2868177 rs13240755	ROS	LVEF decreased	[55]
Huang et al. (2017)	Daunorubicin	ALL	CYP3A5 (POR)	No report	Cytochrome P450 family 3	Cardiac dysfunction	[56]
Vivenza et al. (2013)	Anthracyclines	Breast cancer	GSTM1	No report	Oxidative/electrophilic species	Congestive HF, LVEF	[65]
Rajić et al. (2009)	Anthracyclines	ALL	CAT GSTT1 GSTM1	rs10836235	ROS, SOD	Cardiac damage	[71]
Ruiz-Pinto et al. (2018)	Anthracyclines	Breast cancer	ETFB	rs79338777	Mitochondrial dysfunction	Myocardial injury, LVEF decreased	[74]
Shizukuda et al. (2005)	Doxorubicin	No report	p53	No report	ROS, Cu/Zn, SOD	Cardiac injury, LV systolic dysfunction	[77]
Wang et al. (2014)	Anthracyclines	Children's oncology	HAS3 gene	rs2232228	ROS	LV dysfunction, EF < 40%, and FS < 28%	[79]
Visscher et al. (2015)	Anthracyclines	Childhood cancer	SLC22A17 SLC22A7	rs4982753 rs4149178	ROS, SOD	LV dysfunction	[83]
An et al. (2017)	Doxorubicin	Intermittent fasting	UVRAG	No report	LC3 II and p62 protein	Cardiac dysfunction	[101]
Wang et al. (2018)	Doxorubicin	No report	GCN2	No report	Bcl-2, Bax, ATF4, UCP2	LV dysfunction	[103]
McCaffrey et al. (2013)	Doxorubicin	Breast cancer	TCL1A MDR1	rs11849538	PI3K, AKT, cIAP2, IAP-C, MIHC	Congestive HF, EF < 40%, LV dysfunction	[106]
Todorova et al. (2017)	Doxorubicin	Breast cancer	HLA	rs9264942 rs2523619 rs10484554	Inflammation, autoimmune disorders	Cardiac dysfunction, LVEF decline	[111]
Mori et al. (2010)	Doxorubicin	No report	Spp1 Fhl1 Timp1 Ccl7 Reg3b	No report	Degeneration of myocardium and inflammation	Cardiac injury	[112]
Pop-Moldovan et al. (2017)	Doxorubicin	Hematological malignancies	TLR2 TLR4	No report	TLR	Diastolic dysfunction, LVEF decreased	[118]
Li et al. (2018)	Doxorubicin	Mammary tumor	TLR9	No report	PI3K*γ*	Myocardial dysfunction	[120]
Todorova et al. (2017)	Doxorubicin	Breast cancer	MicroRNA	No report	IL-17, TNF-*α*, NF-*κ*B	Cardiac dysfunction, LVEF declined	[129]
Zhao et al. (2014)	Bevacizumab	Colorectal cancer	miRNA1254	No report	CRP, MMPs	CHF	[132]
Yin et al. (2016)	Doxorubicin	No report	miR-320a	No report	VEGF	Cardiac dysfunction	[135]
Zhu et al. (2017)	Doxorubicin	DLBCL	miR-34a	No report	Caspase-3, Bcl-2	Cardiac dysfunction	[136]
Cascales et al. (2012)	Doxorubicin	Hematological	C282Y-Y H63D-H	No report	Iron metabolism disorder	HF, LVEF decrease	[142]
Lipshultz et al. (2013)	Doxorubicin	ALL	C282Y	No report	Iron metabolism disorder	Cardiac dysfunction, LVEF, cTnT, NT-proBNP	[144]
Ichikawa et al. (2014)	Doxorubicin	No report	ABCB8	No report	Mitochondrial iron	Cardiomyopathy	[146]
Fang et al. (2019)	Doxorubicin	No report	Hmox1	No report	Mitochondrial iron	Cardiomyopathy	[151]
Blanco et al. (2008)	Anthracyclines	Childhood cancer	CBR3 V244M	No report	Metabolism	CHF	[155]
Reinbolt et al. (2016)	Adriamycin, cytoxan	Breast cancer	CBR1 CBR3	No report	Metabolism	EF < 50% and >15%	[156]
Salanci et al. (2012)	Anthracyclines	No report	CBR3 GSTP1	No report	Metabolism	Cardiac dysfunction, LVEFs < 50%	[157]
Blanco et al. (2012)	Anthracyclines	Childhood cancer	CBRs	No report	Metabolism	Cardiomyopathy, EF < 40%, SF < 28%	[158]
Lubieniecka et al. (2012)	Anthracyclines	AML	AKR CBR	No report	Metabolism	LVEF% drop	[162]
Wasielewski et al. (2014)	Anthracyclines	Adult and childhood cancer	MYH7	No report	Sarcomere disruption	Dilated cardiomyopathy	[166]
Wang et al. (2016)	Anthracyclines	Children oncology	CELF4	rs1786814	Sarcomere disruption	Cardiomyopathy	[171]
Garcia-Pavia et al. (2019)	Anthracyclines	Multiple cancers	TTNtv	No report	Sarcomere disruption	Dilated cardiomyopathy	[179]
Ferreira et al. (2017)	Doxorubicin	No report	DNA methylation	No report	Epigenetic	Decreased mtDNA levels	[183]
Ferreira et al. (2019)	Doxorubicin	No report	DNMT1	No report	Epigenetic	Upregulation of mtDNA transcripts	[185]
Beauclair et al. (2007)	Trastuzumab	Breast cancer	Her2	No report	No report	LVEF decreased	[186]
Stanton et al. (2015)	Trastuzumab	Breast cancer	Ile 655 Val Pro 1170 Ala	rs1058808 rs1136201	No report	CHF, LVEF < 50%	[188]
Peña et al. (2015)	Trastuzumab	Breast cancer	HER2 655 A>G	rs1136201	No report	CHF, LVEF < 50%	[189]
Roca et al. (2013)	Trastuzumab	Breast cancer	HER2 Ile655Val	No report	MAPK and PI3 K/Akt	CHF, LVEF < 50%	[190]
Ruiz-Pinto et al. (2017)	Anthracyclines	Pediatric cancer	GPR35	rs12468485	No report	LV dysfunction, SF < 26%	[196]
Sachidanandam et al. (2012)	Doxorubicin	Childhood cancer	HNMT	rs17583889	No report	SF < 26%	[199]
Salata et al. (2013)	Chemotherapy Radiotherapy	Breast cancer	RAS-related genes	No report	AT1 receptor	Cardiac remodeling	[203]
Schneider et al. (2017)	Anthracyclines	Breast cancer	SNP	rs28714259	No report	CHF, LVEF < 50%, acute coronary syndrome, supraventricular tachycardia, myocardial dysfunction	[205]
Kitagawa et al. (2012)	Epirubicin Cyclophosphamide 5-Fluorouracil	Breast cancer	FEC100	No report	No report	Arrhythmias, QTc interval prolongation	[208]

**Table 2:** Protective genes in chemotherapy-induced cardiotoxicity.

Study	Drug used	Type of cancer examined	Gene	ΔExpression	Targets	Cardiac toxicity	References
Krajinovic et al. (2016)	Doxorubicin	ALL	ABCC5 NOS3	No report	ROS	Lower LVEF; reduction of EF and SF	[210]
Zhao et al. (2018)	Doxorubicin	No report	miR-140-5p	Downregulated	ROS	ECG abnormality; histopathological changes of heart	[212]
Fu et al. (2016)	Doxorubicin	No report	GRP78	Upregulated	ER stress	Decreased the LVFS and LVEF	[220]
Hu et al. (2019)	Doxorubicin	No report	CACNA1H	Downregulated	ER stress	Myocardial dysfunction, myocardial apoptosis	[222]
Aung et al. (2017)	Doxorubicin	No report	Mtfp1	Downregulated	ROS, apoptosis	Severe cardiomyopathy	[233]
Kobayashi et al. (2006)	Doxorubicin	No report	GATA4	Upregulated	LC3-II, Bcl-2	Cardiomyocyte death	[240]
Tong et al. (2015)	Doxorubicin	No report	BTG2	Upregulated	Apoptosis, miR-21	Depressed LV function, decreased heart indices	[244]
Chen et al. (2018)	Doxorubicin	No report	CTR P1	Upregulated	PKB/AKT	Impaired cardiac function	[250]
Caso et al. (2017)	Doxorubicin	No report	AKAP-Lbc	Downregulated	Protein kinase D1, Bcl-2, Bax	CytC release and mitochondrial dysfunction	[262]
Chen et al. (2019)	Doxorubicin	No report	SNHG1	Upregulated	miR-195/Bcl-2 axis	Impairment of heart function	[266]
Wells et al. (2017)	Anthracycline	Non-Hodgkin's lymphoma and breast cancer	PRDM2	Upregulated	DNA repair, metabolism, cardiac remodeling	LV function, LVEF	[275]
Zheng et al. (2020)	Doxorubicin	No report	Bnip3	Downregulated	Pyroptosis	Declined in LVEF and FS, increased LDH and CK-MB	[279]
Singla et al. (2019)	Doxorubicin	No report	ES-Exos	Upregulated	Pyroptosis	Cardiac dysfunction	[282]
Dimitrakis et al. (2012)	Doxorubicin	No report	MURF-1	Upregulated	UPS	HF	[287]
Sishi et al. (2013)	Doxorubicin	No report	E3 ligase	Upregulated	UPP	Myocardium dysfunction	[289]
Zhao et al. (2015)	Doxorubicin	No report	*β*1i, *β*2i and *β*5i	Upregulated	UPS	Cardiac dysfunction	[290]
Chen et al. (2015)	Adriamycin	No report	MG132	Upregulated	Cx43, ZO-1, 20S proteasome	HF	[295]
Spur et al. (2016)	Doxorubicin	No report	LMP7	Downregulated	b5 standard proteasome	HF	[296]
Song et al. (2018)	Doxorubicin	No report	*α*-Tubulin acetylation	Downregulated	HDAC6	Acute cardiomyopathy	[302]
Hanf et al. (2019)	Doxorubicin	No report	Histone 3 acetylation	Downregulated	SIRT1 and HDAC2	Cardiomyopathy	[303]
Piotrowska et al. (2017)	Doxorubicin	No report	Anf, Bnf, Bmp-1, Myh-7	Upregulated	HDACs	Cardiac remodeling	[307]
Bhuvanalakshmi et al. (2017)	Doxorubicin	No report	sFRP4	Upregulated	HDACs	Cardiac injury	[308]
Räsänen et al. (2016)	Doxorubicin	No report	VEGF-B	Upregulated	Apoptosis	Decreased LV mass, left ventricular wall and septum thickness, diastolic and systolic volume, and stroke volume; decreased LVFS and LVEF	[313]
